# Use of mobile phone during pregnancy and the risk of spontaneous abortion

**DOI:** 10.1186/s40201-015-0193-z

**Published:** 2015-04-21

**Authors:** Fatemeh Shamsi Mahmoudabadi, Saeideh Ziaei, Mohammad Firoozabadi, Anoshirvan Kazemnejad

**Affiliations:** Faculty of Medical Science, Tarbiat Modares University, Tehran, Iran; Reproductive Health Department, Tarbiat Modares University, P. O. Box: 14115–111, Tehran, Iran

**Keywords:** Abortion, Electromagnetic fields, Mobile phones, Pregnancy

## Abstract

**Background:**

Exposure to electromagnetic fields of cell phones increasingly occurs, but the potential influence on spontaneous abortion has not been thoroughly investigated.

**Methods:**

In a case–control study, 292 women who had an unexplained spontaneous abortion at < 14 weeks gestation and 308 pregnant women > 14 weeks gestation were enrolled. Two data collection forms were completed; one was used to collect data about socioeconomic and obstetric characteristics, medical and reproductive history, and lifestyles. Another was used to collect data about the use of cell phones during pregnancy. For the consideration of cell phone effects, we measured the average calling time per day, the location of the cell phones when not in use, use of hands-free equipment, use of phones for other applications, the specific absorption rate (SAR) reported by the manufacturer and the average of the effective SAR (average duration of calling time per day × SAR). Analyses were carried out with statistical package state software(SPSS)v.16.

**Results:**

All the data pertaining to mobile phones were different between the two groups except the use of hands free devices (p < 0.001).

**Conclusion:**

Our result suggests that use of mobile phones can be related to the early spontaneous abortions.

## Introduction

Widespread concerns have been raised about exposure to electromagnetic field (EMF) from sources used for mobile telecommunication [1-4]. Much of the concern arises because new technologies are introduced without adequate provision upon public information about their nature or discussion of the debate within the scientific community about possible health consequences. Indeed, mobile phone use has increased considerably along with reducing its costs, and developing countries are establishing mobile telecommunications rather than the more expensive fixed-line systems. Thus, if there is an impact on health from mobile telephones, it will affect everyone in the world. The major focus of research has been on radio-frequency (RF) radiation, which is mainly generated by phones, while some scientists are concerned about the possible impact of exposure to extremely low frequency electromagnetic field (ELF-EMF) fields generated by supply currents in the phone [5-7]. The device with the largest power consumption is the front-end amplifier. Consequently, the corresponding ELF-EMF has a spectrum similar to the pulse structure of RF signals.

The public discussion of the health hazards of EMF exposure has focused on the possible association with cancer, cardiovascular and immune systems. Less attention has been paid to the evaluation of evaluating the health hazards of EMF exposure on reproductive health such as spontaneous abortions [7-9].

The health effect of EMF on pregnancy has remained controversial despite efforts to reach consensus [10]. The main challenges in studying EMF are; 1) accurately assessing during the relevant time period and; 2) identifying susceptible population.

Therefore a case–control study has been designed o determine the effects of EMF exposure to mobile phones immediately after spontaneous abortions in selected patients.

## Methods

For the evaluation of the effects of EMF exposure to mobile phones on spontaneous abortions, a case control study was chosen. Due to the lack of knowledge about these effects, a pilot study on 100 participants was carried out. Then, based on the primary data, with a significance level of α = 0.05 and power of 1-β = 0.80,a sample size of 220 participants for each group was calculated. In the current case–control study, 292 women who had an unexplained spontaneous abortion at < 14 weeks gestation (as early abortion) immediately after abortion(during first 48 hours) and 308 pregnant women at 14 weeks gestation were enrolled. Gestational age was calculated based on the last menstrual period that was confirmed by an ultrasonographic examination. The subjects were recruited from 10 hospitals in Tehran.

Pregnancy situations in the pregnant women in the control group were matched with maternal and paternal ages, gravidity, pre-pregnancy BMI, family relationship, duration from last delivery, educational level, occupation and history of previous abortions or preterm labor with the women in the case group.

The inclusion criteria were as follows:

1. singleton pregnancy; 2) 18–35 years of age; 3) spontaneous pregnancy without the use of assisted reproductive technology

The exclusion criteria were as follows:

1. chronic diseases (diabetes, hypertension,thyroid diseases, immune system diseases and cardiovascular diseases); 2) any genetic disorders (in the women, their husbands,and their first-degree relatives);3) vaginal bleeding in the first trimester of pregnancy in the control group; 4) history of birth defects in previous pregnancies; and 5) cigarette smoking, alcohol or any drug consumptions.

The women completed the data collection form, which was used to collect data about socioeconomic and obstetric characteristics, medical and reproductive histories, and lifestyles. The second data collection form which was completed by trained interviewer was designed to obtain information about the use of cell phones during pregnancy. For the consideration of cell phones effects, we measured the average calling time per day, the location of the cell phones when not in use (in handbags, clothing pockets, or ≥60-70 cm away from the body), use of hands-free equipment, use of phones for other applications (to send messages, to listen to music, and to play games), the specific absorption rate (SAR) reported by the manufacturer and the average of the effective SAR (average duration of calling time per day × SAR). SAR indicates how much electromagnetic energy is absorbed by body tissues. This parameter shows the estimation of total EMF energy. The effective SAR can show the exposure of ELF estimation from mobile phones indirectly. We investigated the data about the SAR of each mobile phone model from SAR.ir site.

For precise evaluation of the calling time, the receipts of mobile phone bills were considered. The information about the time spent for telecommunication in the specific section of new versions of cell phones were also taken into consideration.

The procedure was approved by the Ethics Committee of Tarbiat Modares University. All of the women participated voluntarily and provided written informed consent.

### Statistical analysis

Demographic and obstetric characteristics, as well as the parameters which reflect the use of cell phones were compared between the two groups using t-tests and chi-square tests. A logistic regression model was used to assess the association between spontaneous abortions as a binary dependent factor and effective SAR, maternal age, paternal age, history of abortion and family relationship as independent factors. Analyses were carried out with statistical package state software(SPSS)v.16.A P-value <0.05 was considered statistically significant. All confidence intervals were calculated at the 95% level.

## Results

The distribution of demographic and potential confounders for spontaneous abortions is presented in Table 1. As shown, there were no significant differences in maternal and paternal ages, pre-pregnancy BMI, occupation and educational status, socioeconomic level, family relationship, duration from last delivery and history of previous abortions or preterm labors between the two groups.

**Table 1:** **The comparison of the maternal and paternal characteristics between the two groups**

**Parameters**	**Case group(n:226)**	**Control groups(n:246)**	**P**
Maternal age(years)* Mean ± SD	27.81 ± 5.20	27.34 ± 4.3	0.2
Paternal age(years)* Mean ± SD	32.62 ± 5.46	31.90 ± 5.49	0.1
Family relationship** N(%)	62(21.24)	77(25.1)	0.3
Pre-pregnancy BMI*	25.14 ± 6.98	24.29 ± 5.24	0.09
History of abortion** N(%)	47(20.8)	36(14.6)	0.2
History of preterm labor**N(%)	3(1.3)	2(0.8)	0.6
Duration from last delivery(years)* Mean ± SD	6.61 ± 3.93	6.57 ± 3.46	0.9
Occupation N(%)**			0.2
Housewife	195(86.4)	225(91.5)	
Employee	31(13.6)	21(8.5)	
Educational status N(%)**			0.09
Primary school	88(38.9)	98(39.8)	
Secondary and high school	94(41.6)	100(40.6)	
University	44(19.5)	48(19.6)	

*T student test **Chi square test.

As shown in Table 2, all of the data pertaining to the use of cell phones such as average calling time per day, the location of the cell phones when not in use, use of phones for other applications, the specific absorption rate (SAR) and the average of the effective SAR), except the use of hands free devices were different between the two groups(p < 0.001). Logistic regression analysis revealed a significant association between the effective SAR with the risk of spontaneous abortions after adjustment for maternal age, paternal age, history of abortions and family relationships (OR:1.11,p < 0.001) (Table 3).

**Table 2:** **The comparison of cell phone use during pregnancy between the two groups**

**Parameters**	**Case group(n:226)**	**Control group(n:246)**	**P(2-tailed)**
Calling time per day* (minutes) Mean ± SD	9.31 ± 15.22	3.14 ± 3.59	<0.001
Use of hands free** n(%)	19(8.4)	11(4.5)	0.09
location of phones when not in use** n(%)			<0.001
on tables	86(38.1)	77(31.3)	
in handbags	101(44.7)	153(62.2)	
in pocket	39(17.3)	16(6.5)	
use of phone for other applications **n(%)	90(39.8)	44(17.9)	<0.001
Effective SAR* Mean ± SD	7.02 ± 13.92	2.28 ± 3.20	<0.001

*T student test, ** Chi square test or Fisher’s exact test,SAR: Specific Absorption Rate.

**Table 3:** Association of spontaneous abortions with the effective SAR(Specific Absorption Rate) *

	**OR(95%CI)**	**B**	**SE**	**P**
Effective SAR	1.11(1.07-1.16)	0.106	0.21	<0.001

*Adjusted for maternal age, paternal age, history of abortion and the spouse relatives.

## Discussion

Exposure to EMF is increasingly common, but the potential influence on health has not been thoroughly determined, especially in pregnant women. Electromagnetic fields may produce biological stress and free radicals, which can make a susceptible population prone to congenital malformations and tissue and cell damages [11]. Long-term exposure to electromagnetic fields may be linked to even higher levels of oxidative stress, with the aforementioned corresponding changes.

There is limited information on the association between EMF exposure during pregnancy and reproductive outcomes [10-18]. Some studies have reported increased risk of spontaneous abortions and congenital malformations, although these results were derived from poorly designed studies.

This study demonstrated an increased risk of spontaneous abortions associated with EMF exposure and confirmed the results of other researches [10,12]. Lee conducted a nested case–control study to evaluate the association of residential and personal magnetic fields with spontaneous abortions [12]. He reported personal magnetic field maximum exposure is associated with the risk of clinical spontaneous abortions. There was one main limitation to that study; specifically, the magnetic field measurements were obtained months after the first trimester and the occurrence of the spontaneous abortions. Perhaps the behavior and magnetic fields of women who had spontaneous abortions were different from the pregnant controls. But our study had this strength that, the data were obtained shortly after the spontaneous abortions occurred in the case group and in the early second trimester in the control group. This led to a better evaluation of the environmental conditions and the lifestyles of the participants

Though, the present findings are inconsistent with the results of some other works [14-18]. Some studies do not support an association between electric and magnetic fields and reproductive damages. However, many of them had investigated the effects of EMF either on animals or in vitro and not on human [14-16]. Also, lack of evidence for a strong association between EMF and adverse reproductive outcomes may be primarily the result of having little information data due to the lack of epidemiologic data in this area.

One potential limitation need to be kept in mind when one interprets the results of the current study. The study did not assess all the unknown risk factors for spontaneous abortions, such as balanced chromosomal abnormalities in one parent. Also, the data about unknown spontaneous abortion at very early stages were not collected. Other limitation of this study is its case control nature, and this can imply that caution should be taken in causal interpretations of the findings. The third limitation of this study is that cell phones may be not the only source of EMF. Since many parameters which can affect the EMF exposure and life styles like socioeconomic status were matched between the two groups, it is suspected that probably the effects that observed in this study are due to the use of cell phones.

The association between use of cell phones and the risk of spontaneous abortions against potential confounders was supported by evidence that despite adjustments for many known or suspected risk factors in logistic regression analyses, the estimation was not significantly altered.

Although the mechanisms underlying the effects of EMF on the risk of spontaneous abortions are not well understood, early embryos are known to be sensitive to environmental exposures. An adverse effect during early fetal development at the cellular level by EMF of cell phones could conceivably result in fetal death. EMF in cell phones is both ELF-EMF and RF-EMF. Based on the distance from outside the body to inside the uterus, the exposure reaching the fetus is likely to be extremely low frequency electromagnetic radiation.

## Conclusion

Despite the lack of a clear understanding of the underlying mechanisms, the present result suggests that the use of cell phones may be related to early spontaneous abortions, thus further study is warranted.
